# Soil-like Substrate Technology Improves Soil Nutrient Content and Enzyme Activity, Enhancing Soil Microbial Community Structure and Restoring Soils in Ecologically Sensitive Areas of the Loess Plateau

**DOI:** 10.3390/microorganisms13112621

**Published:** 2025-11-19

**Authors:** Gexue Bai, Qingqing Tan, Bingbing Han, Ruidong Li, Lijun Gu, Xiaojing Wang, Yan Li, Quanfang Zhang

**Affiliations:** 1 Gansu Institute of Engineering Geology, Lanzhou 730000, China; baigexue6009@163.com (G.B.); 13519653867@163.com (B.H.); 13919456599@163.com (R.L.); 2 Institute of Crop Germplasm Resources, Shandong Academy of Agricultural Sciences, Jinan 250100, China; tanqingqing88@163.com; 3 College of Pastoral Agriculture Science and Technology, Lanzhou University, Lanzhou 730020, China; gulj@lzu.edu.cn; 4 Institute of Crop Research, Tianjin Academy of Agricultural Sciences, Tianjin 300381, China; 210-wxj@163.com; 5 State Key Laboratory of Nutrient Use and Management, Shandong Academy of Agricultural Sciences, Jinan 250100, China

**Keywords:** soil-like substrate technology, nutrient content, enzyme activity, microbial community structure, functional annotation, correlation

## Abstract

The study assessed the impact of soil-like substrate technology on soil nutrient cycling, enzyme activities, and microbial community structure to evaluate its potential for ecological restoration in the highly sensitive areas of the Loess Plateau. Soil nutrients and enzyme activities were measured before and after applying the technology and at various soil depths. Microbial diversity and community structure were analyzed using Illumina PE150 sequencing. In the −20 cm depth layer (RLS), soil nutrient content and enzyme activity were significantly higher than in the control (CK). Compared with CK, total nitrogen and organic matter in RLS increased by 1.35 and 1.03 times, respectively. Urease and invertase activities increased by 1.15 and 1.35 times, respectively. Microbial community analysis showed changes in *Actinomycetes*, *Alphaproteobacteria*, and *Thermoleophilia* populations. The surface layer (0–6 cm, SS) had higher nutrient content and enzyme activity than deeper layers. The microbes in the SS layer were significantly different from those in the substratum layer (6–12 cm, BS) and the vegetation mat substrate layer (12–20 cm, PS). The top three most abundant phyla were *Nocardioidaceae*, *Micrococcaceae*, and *Unclassified-Frankiales*. Kyoto Encyclopedia of Genes and Genomes (KEGG) analysis indicated that microbes in the surface layer were mainly involved in carbohydrate and amino acid metabolism. Correlation analysis revealed significant relationships between environmental factors and microbial communities. Soil-like substrate technology enhances soil nutrients, enzyme activity, and microbial community structure, providing evidence for restoring the “soil-vegetation-microorganism” system in the Loess Plateau.

## 1. Introduction

The soil-like substrate is an innovative soil improvement technology that aims to simulate the structure and function of natural soil to enhance soil quality and promote vegetation recovery. This technology was first proposed by Russian researchers Manukovsky et al. [1]. The Loess Plateau, as an ecologically vulnerable area, has been exploring effective soil erosion control methods, providing the background and demand for the application of new technologies such as slope repair [2]. Natural vegetation restoration has become an effective and rapid method for ecological recovery in vulnerable areas [3], and different vegetation types can affect soil aggregates, organic carbon, and nitrogen [4], as well as the mutual response between soil microorganisms and vegetation succession [5,6,7]. Therefore, the development and application of soil-like substrate, as an innovative soil improvement technology, are in line with the ecological recovery needs of the Loess Plateau. It helps to improve the soil environment, thereby affecting the structure and function of soil microbial communities, and has shown significant potential in areas with severe soil erosion, especially in ecologically vulnerable regions like the Loess Plateau in China. The development of soil-like substrate includes the careful selection and combination of organic and inorganic materials to enhance soil properties and support plant growth. Its components usually include organic materials such as plant residues of rice and wheat straw, spent substrates from mushroom cultivation, and organic matter processed by earthworms, as well as inorganic materials such as mineral particles, volcanic soil, vermiculite, and perlite. These components, through the action of specific microorganisms (such as nitrogen-fixing bacteria and mycorrhizal fungi), promote the decomposition of organic matter and the weathering of minerals, forming organic matter-mineral complexes, thereby improving soil structure. In severely eroded soils, the application of soil-like substrate technology significantly improves the physical properties of the soil by increasing soil porosity, enhancing water retention, and aeration [8]. In addition, the soil-like substrate provides good conditions for the colonization of pioneer plants, improves soil nutrient balance, and supports plant growth [9]. This technology not only increases soil fertility but also enhances the ecological functions of the soil by promoting the formation of microbial communities.

Currently, academia commonly employs three major indicator systems to comprehensively evaluate soil health: soil physicochemical properties, enzyme activity, and microbial diversity. The nutrient content of physicochemical indicators (including pH, available phosphorus, available nitrogen, organic carbon, and total nitrogen) influences vegetation growth potential. Soil enzyme activity characterizes biochemical process intensity across different dimensions: urease governs nitrogen cycling, phosphatase drives phosphorus transformation, invertase regulates carbon source metabolism, and catalase reflects the antioxidant defense level of microbial communities. These three types of indicators maintain the stability and functional integrity of the soil ecosystem through a complex network of interactions. With the rapid development of molecular biology technologies, the use of advanced techniques such as high-throughput sequencing to reveal the dynamic interactions between soil physicochemical properties, enzyme activities, and microbial diversity has become a cutting-edge research focus in current soil ecology [10,11,12]. This research direction provides new technological pathways and theoretical perspectives for a deeper understanding of the functioning mechanisms of soil ecosystems.

The Loess Plateau, as the world’s largest loess accumulation area [13], is not only a core region of the “Belt and Road Initiative”, but also a key area for the Western Development Strategy. However, the region has long faced serious problems such as soil erosion, vegetation degradation, and ecological fragility [13], which have damaged the structure of soil microbial communities and affected the stability of ecosystems [14]. Although previous studies have focused on vegetation restoration [15], soil environment [16,17], microbial diversity [18], hydrological characteristics [19,20,21], and carbon emissions [22,23], research on the synergistic effects of “plants-soil microbes-soil physicochemical properties” remains relatively weak [24,25]. In the upper reaches of the Yellow River in Gansu, severe soil and water loss has been exacerbated by human engineering activities (such as the excavation of steep slopes), making natural recovery extremely difficult. Soil-like substrate technology can effectively improve soil structure and promote vegetation restoration, but its regulatory mechanisms on soil microbial-nutrient cycling-plant growth are still unclear. Soil microorganisms play a key role in nutrient cycling, plant growth, and ecosystem stability. Therefore, understanding how Soil-like substrate affects the structure and function of soil microbial communities is crucial for assessing its long-term effectiveness in ecological restoration.

This study primarily investigates the interrelationships among soil nutrient content, enzyme activity, and microbial community structure before and after the application of soil-like substrate technology, including variations across different soil depths. The research area is located in Gaolan Mountain, Lanzhou City, Gansu Province. This study employs the methods introduced in the third edition of “Soil and Agricultural Chemical Analysis” by Bao S.D. to detect soil nutrient content and uses a spectrophotometer to detect relevant enzyme activities [26]. Meanwhile, the Illumina PE150 sequencing platform was used to analyze the soil microbial community structure and functional annotation. This study aims to verify the following hypotheses: (1) soil-like substrate technology can significantly increase soil nutrient content, thereby enhancing soil enzyme activity and microbial diversity, and revealing the intrinsic correlations among the three; (2) Microbial communities exhibit significant differences in composition and metabolic functions across different soil depths in the soil-like substrate technology treatment area, with higher biological activity observed in the surface soil.

## 2. Materials and Methods

### 2.1. Study Site

The experimental site is located on the east side of Laolang Gully in Gaolan Mountain, Lanzhou City, Gansu Province, China, at an elevation of 2021 m (36.0193° N, 103.8495° E). The area has a temperate semiarid climate, with an average annual temperature of 7.2 °C, average annual precipitation of 266 mm, annual sunshine duration of 2768 h, and a frost-free period of 144 days. The soil nutrient contents are as follows: total nitrogen 4.1 g·kg^−1^, available nitrogen 25.0 mg·kg^−1^, available phosphorus 8.1 mg·kg^−1^, available potassium 11.12 mg·kg^−1^, organic matter content 6.81 g·kg^−1^, and pH 7.92. The experimental site is in the second year of treatment with soil-like substrate technology, with vegetation mainly consisting of herbaceous plants and shrubs. The main arbor species are *Platycladus orientalis* and *Salix matsudana*.

### 2.2. Erosion Control System

The treatment of soil-like substrate technology and the addition of organic materials can improve the physical structure of the soil, enhancing its aeration and water retention capabilities. After one year of monitoring, the vegetation coverage in the study area reached 75%, with 22 different plant species. These plants grew rapidly under the soil and climatic conditions of the study area, forming a dense vegetation cover that effectively reduced the direct impact of rainwater on the soil. On steeper slopes, terraces were constructed. Additionally, a comprehensive drainage system was installed along the edges and in the low-lying areas of the study area. Soil erosion monitoring stations were established to regularly collect data and assess the degree of erosion.

### 2.3. Soil Sampling

**Figure 1:**
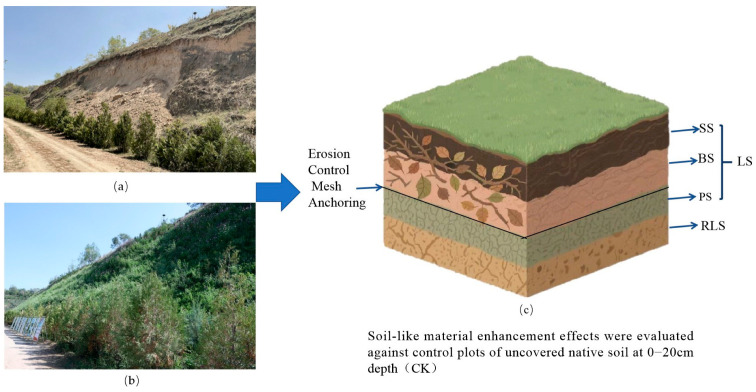
Schematic diagram of the soil-like substrate technology restoration and soil sampling depths in the experimental area. (**a**) Before soil-like substrate modification, (**b**) After soil-like substrate modification; (**c**) Schematic diagram of sampling depths in the experiment. First, different experimental plots were delineated. Subsequently, soil samples were sequentially collected at different depths using a graduated tube soil auger in accordance with the five-point sampling method.

In this experiment, the main components of the soil-like substrate are organic matter (30–40%), binder (<1%), and straw fiber (2–3%), which are then mixed with soil at a ratio of 0.1% and subjected to technical treatment on a slope. The area without soil-like substrate coverage at 0–20 cm depth was used as the control zone (CK), and the area at −20 cm depth beneath the soil-like substrate (RLS) was used as the experimental zone to study the soil restoration characteristics of soil-like substrate technology (Jiangsu Green Rock Ecological Technology Co., Ltd., Zhangjiagang, China; Gansu Gansu Lanshan Cloud Ecological Technology Co., Ltd., Lanzhou, China) in the ecologically sensitive areas of the Loess Plateau. Additionally, soil samples were taken from different depths within the soil-like substrate technology zone (LS), namely the surface layer (0–6 cm, SS), the Substratum layer (6–12 cm, BS), and the vegetation mat substrate layer (12–20 cm, PS), to further analyze the soil improvement mechanisms of the soil-like substrate technology. In the subsequent data analysis, Arabic numerals will be used after the abbreviations to indicate replicates. For example, CK1, CK2, CK3, and RLS1, RLS2, RLS3 represent the three replicates of CK and RLS, respectively. The study area was divided into three experimental plots, each with an area of 10 × 10 m^2^. In each experimental plot, samples were collected using the five-point sampling method, mixed evenly, and then divided into three replicates for testing [27]. For samples from different sampling depths, the depth of the graduated tube soil auger was adjusted according to the information on soil-like substrate technology treatment, and sampling was carried out in sequence. The samples were collected, with surface debris such as branches and dead grass removed. The samples were then mixed thoroughly, sieved, and used for the determination of soil nutrient content and enzyme activity, as well as for metagenomic analysis. The sampling schematic is shown in Figure 1.

### 2.4. Determination of Soil Nutrient Content and Enzyme Activity

The collected soil samples were analyzed for nutrient content and enzyme activity. Total nitrogen (TN) in the soil was determined using continuous flow analysis. Hydrolyzable nitrogen (HN) was measured by the alkali hydrolysis diffusion method, available phosphorus (AP) by the molybdenum–antimony colorimetric method, and available potassium (AK) by flame photometry. Soil organic matter (SOM) was determined by the potassium dichromate external heating method. pH and electrical conductivity were measured in an aqueous solution after mixing 10 g of soil with 25 mL of water [27]. The activities of soil enzymes, including urease (UE), catalase (CAT), alkaline phosphatase (ALP), and invertase (IA), were determined using the working solution prepared according to the kit instructions. (Beijing Box Biotech Co., Ltd., Beijing, China). The detection of enzyme activity is performed according to the specific instructions provided in the corresponding kit, and then the enzyme activity is measured using a UV-visible spectrophotometer (Suzhou Shimadzu Instruments Co., Ltd., Suzhou, China) at different wavelengths.

### 2.5. Analysis of Microbial Community Structure Based on High-Throughput Sequencing

Genomic DNA was extracted from soil samples using a commercial kit (E.Z.N.A.^®^ Soil DNA Kit, Omega Bio-Tek, Norcross, GA, USA). The purity and integrity of the DNA were analyzed by agarose gel electrophoresis, and the DNA concentration was accurately quantified using a Qubit fluorometer (Thermo Fisher Scientific, Waltham, MA, USA). Qualified DNA samples were randomly fragmented into approximately 350 bp fragments using a Covaris ultrasonicator (Covaris, Woburn, MA, USA). The library preparation was completed through a series of steps, including end repair, A-tailing, adapter ligation, purification, and PCR amplification. After library construction, the DNA concentration was initially quantified using Qubit 2.0, and the library was diluted to 2 ng/µL. The insert size of the library was then detected using an Agilent 2100 Bioanalyzer (Santa Clara, CA, USA). If the insert size met the expected range, the effective concentration of the library was accurately quantified using qPCR (library effective concentration > 3 nM) to ensure library quality. After passing the quality check, the library was sequenced on the Illumina PE150 platform (San Diego, CA, USA) for metagenomic analysis.

### 2.6. Statistical Analysis

Raw data obtained from the Illumina HiSeq sequencing platform were preprocessed using Readfq (https://github.com/cjfields/readfq, accessed 18 August 2023) to obtain clean data for subsequent analysis. Subsequently, the assembled scaffolds were broken at the N connection points to obtain scaffolds without N. The clean data were then assembled using the MEGAHIT software (https://github.com/voutcn/megahit, accessed 18 August 2023). The assembled scaffolds (≥500 bp) from each sample were subjected to ORF prediction using MetaGeneMark (http://topaz.gatech.edu/GeneMark/, accessed 18 August 2023). For the ORF prediction results, the CD-HIT software (http://www.bioinformatics.org/cd-hit/, accessed 18 August 2023) was used to remove redundancy in order to obtain a non-redundant initial gene catalog. Bowtie2 was then used to align the clean data from each sample to the initial gene catalog, calculating the number of reads aligned to each gene in the samples. Based on the abundance information of each gene in the gene catalog across samples, basic information statistics, inter-sample correlation analysis, and Venn diagram analysis of gene numbers were performed. The sequences were aligned with database information using DIAMOND software (https://github.com/bbuchfink/diamond/, accessed 19 September 2023), and species annotation information was determined using the MEGAN software’s (https://en.wikipedia.org/wiki/Lowest_common_ancestor, accessed 19 September 2023) systematic classification. Starting from the abundance tables at various taxonomic levels, Principal Component Analysis (PCA) was performed using the R package ade4 to reduce dimensionality and maximize the representation of differences between and within samples on two coordinate axes. Additionally, one-way (ANOVA) analysis of variance and multiple comparisons (LSD) were conducted using SPSSPRO software (https://www.spsspro.com, accessed 19 September 2023). Correlation analysis was performed using the R4.4.2 and Origin 2022.

## 3. Results

### 3.1. Effects of Soil-like Substrate Technology on Soil Nutrient Content and Enzyme Activity

**Figure 2:**
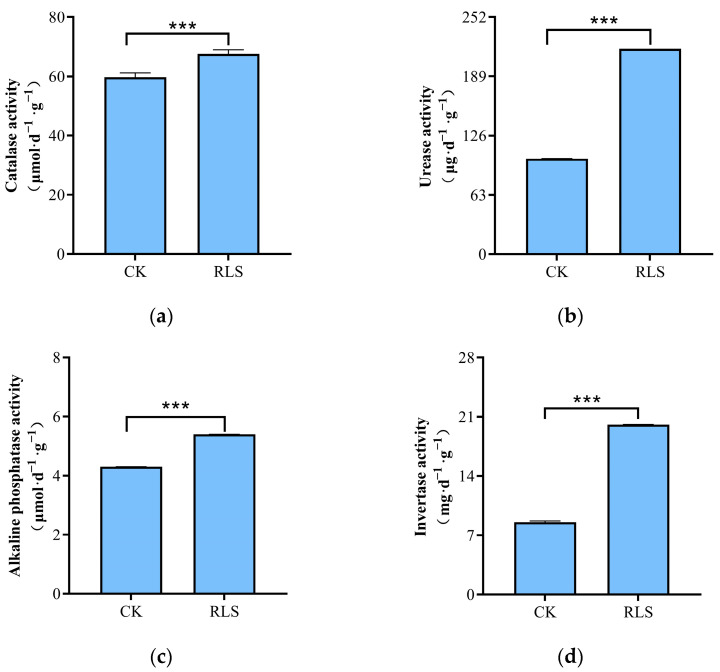
Enzyme activities in control and treatment areas. (**a**) Catalase activity; (**b**) Urease activity; (**c**) Alkaline phosphatase activity; (**d**) Invertase activity. Asterisks denote significance levels: *** *p* < 0.001.

The results of soil nutrient content and enzyme activity in CK and RLS treatments showed that the contents of TN, HN, AP, AK, SOM, and pH in RLS soil samples were significantly higher than those in CK, with increases of 1.35 times, 1.31 times, 55.70%, 67.41%, 1.03 times, and 6.01%, respectively. The EC was lower than CK (*p* < 0.05), with a decrease of 82.49%. The activities of UE, CAT, ALP, and IA were significantly higher than those in CK, with increases of 1.15 times, 13.14%, 25.44%, and 1.35 times, respectively (Table 1 and Figure 2a–d).

### 3.2. Effects of Soil-like Substrate on Soil Microorganisms

#### 3.2.1. Principal Component Analysis of Soil-like Substrate

**Figure 3:**
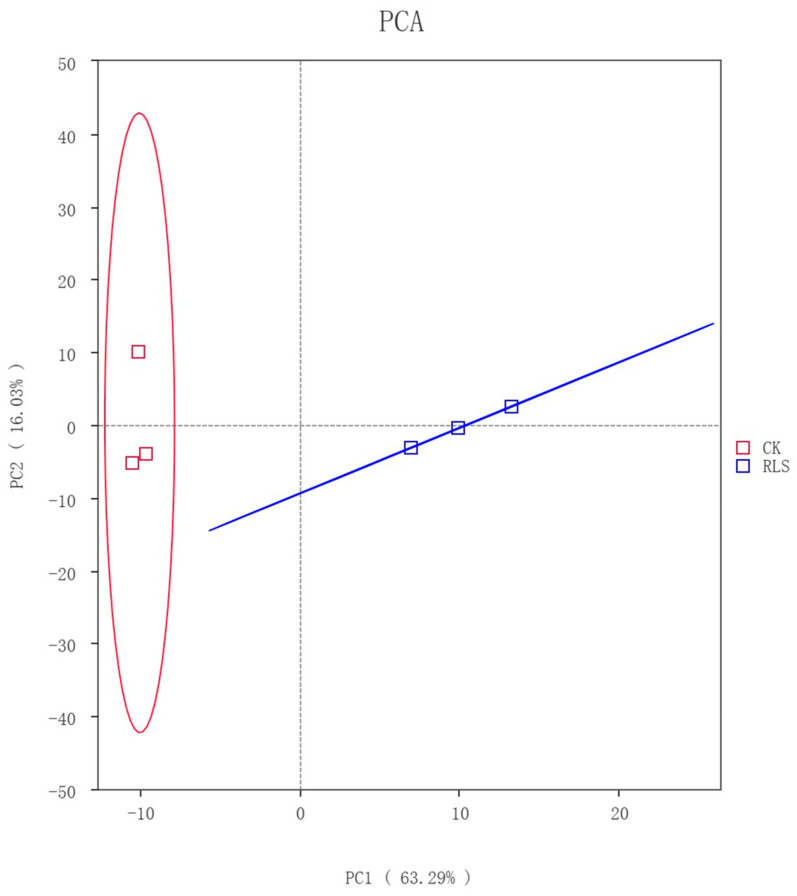
Principal Component Analysis (PCA) of Soil-like Material Matrix.

Principal Component Analysis (PCA) of CK and RLS samples revealed that the first principal component (PC1) accounted for 63.29% of the variance, while the second principal component (PC2) accounted for 16.03% of the variance, with a cumulative contribution rate of 79.32% (Figure 3). The two samples clustered into distinct groups, with a considerable distance between them, indicating significant differences in the microbial community structures of CK and RLS.

#### 3.2.2. Characteristics of Soil Community Changes in Soil-like Substrate

**Figure 4:**
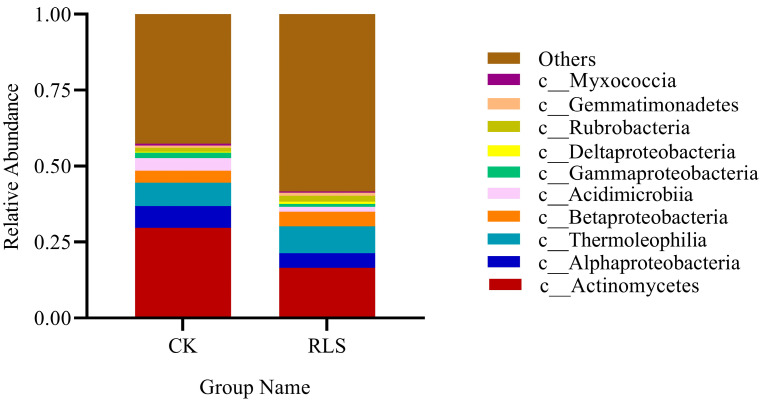
Column chart of the relative abundance of soil microbial community structures in CK and RLS. The others are all microorganisms with an abundance below ten, including both identified and unidentified taxonomic units.

**Figure 5:**
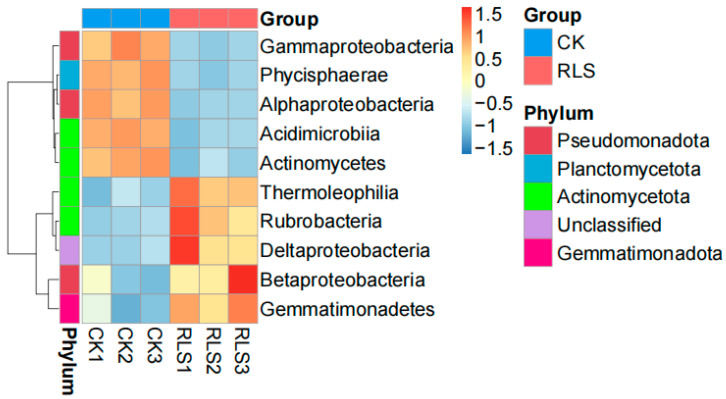
Heatmap illustrating the top 10 class-level abundances of soil microorganisms. CK1, CK2, and CK3, as well as RLS1, RLS2, and RLS3, represent the three replicates of CK and RLS, respectively.

At the class level, the bacterial community structures of CK and RLS were similar, but there were significant differences in relative abundance (Figure 4). Among the top ten most abundant phyla, the main phyla with relative abundance greater than 5% in both samples were *Actinomycetes*, *Alphaproteobacteria*, and *Thermoleophilia*. Compared to CK, the relative abundance of *Actinomycetes* and *Alphaproteobacteria* in RLS decreased by 44.45% and 31.34%, respectively. Cluster analysis based on differences in phylum and class-level species abundance revealed variations in microbial diversity (Figure 5). Compared to CK, RLS had higher relative abundances of *Thermoleophilia* (increased by 13.29%), *Rubrobacteria* (increased by 37.19%), *Deltaproteobacteria* (increased by 54.48%), *Betaproteobacteria* (increased by 15.97%), and *Gemmatimonadetes* (increased by 27.66%). The percentages are shown in Table 2.

### 3.3. Soil Nutrient Content and Enzyme Activity in Soil-like Substrate at Different Depths

**Figure 6:**
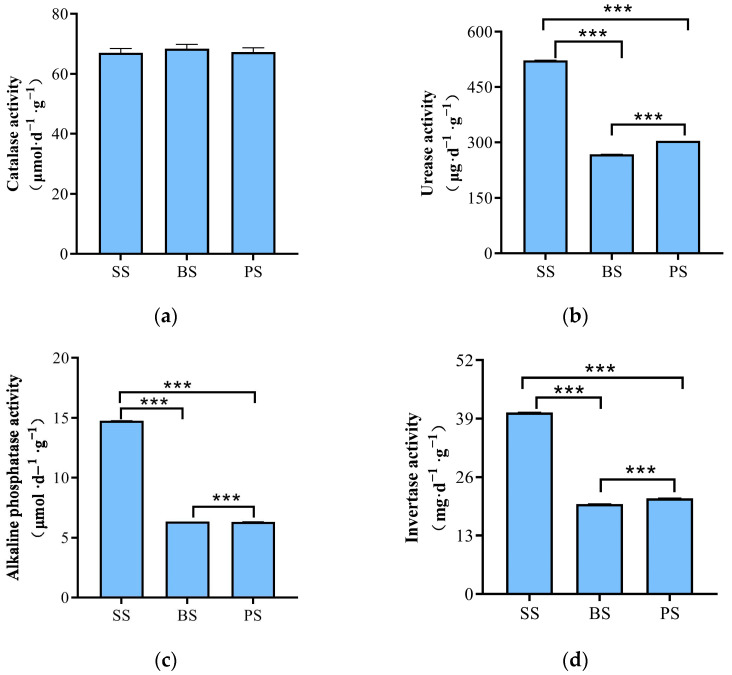
Enzyme activity at different depths in the soil-like substrate technology zone. (**a**) Catalase activity; (**b**) Urease activity; (**c**) Alkaline phosphatase activity; (**d**) Invertase activity. Asterisks denote significance levels: *** *p* < 0.001.

Soil samples from the soil-like substrate technology zone (LS) were analyzed for nutrient content and enzyme activity at different depths. The results showed that the contents of AP, HN, SOM, TN, and AK in the SS layer were higher than those in the BS and PS layers, with values of 79.9 mg·kg^−1^, 99.38 mg·kg^−1^, 22.40 g·kg^−1^, 0.129%, and 27.64 mg·L^−1^, respectively (Table 3). There were no significant differences in CAT enzyme activity among the different soil depths. The activities of UE, ALP, and IA in the SS layer were significantly higher than those in the BS and PS layers. Specifically, UE activity increased by 95.29% and 71.76% compared to BS and PS, respectively; ALP activity increased by 1.32 and 1.33 times compared to BS and PS, respectively; and IA activity increased by 1.01 times and 89.35% compared to BS and PS, respectively (Figure 6a–d).

### 3.4. Analysis of Soil Microbial Diversity at Different Depths of Soil-like Substrate

#### 3.4.1. Principal Component Analysis of Soil Microbes at Different Depths of Soil-like Substrate

**Figure 7:**
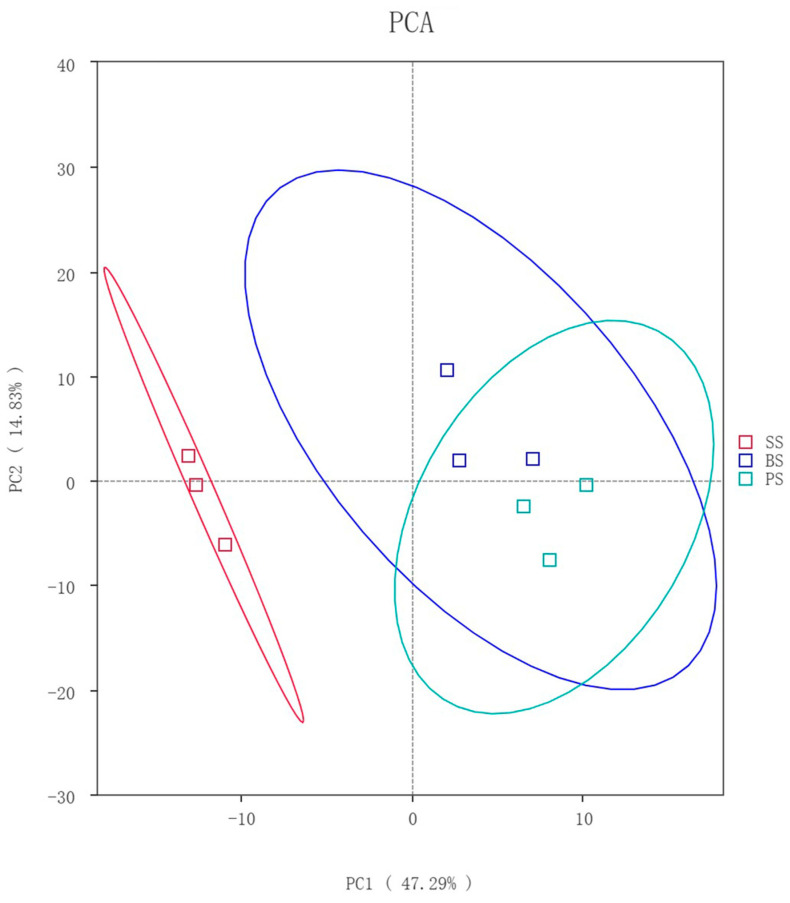
Microbial PCA at different depths in the soil-like substrate technology zone.

The Principal Component Analysis (PCA) results showed that PC1 and PC2 explained 47.29% and 14.83% of the sample composition, respectively, with a cumulative contribution rate of 62.12%. The BS and PS samples were closer to each other, indicating smaller community differences, while the SS sample was more distant, showing differences in microbial community structure (Figure 7).

#### 3.4.2. Analysis of Alpha Diversity Indices of Soil Microbes at Different Depths of Soil-like Substrate

**Figure 8:**
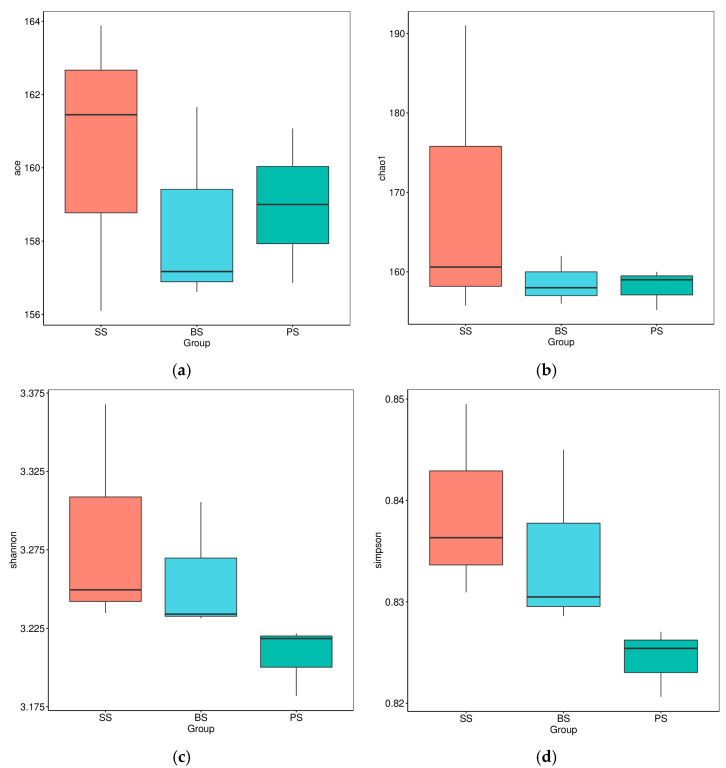
Alpha diversity indices of soil microbes at different depths in the soil-like substrate technology zone. (**a**) ace indices; (**b**) chao1 indices; (**c**) shannon indices; (**d**) simpson indices.

Alpha diversity indices are important indicators for assessing soil microbial diversity. The coverage of the constructed libraries reached over 99%, indicating that the libraries established in this study effectively reflect the diversity of soil microbial communities. As shown in Figure 8, the chao1, shannon, and simpson indices of the SS samples were higher than those of the BS and PS samples. The ace index in the BS soil samples was slightly higher than that in the SS and PS samples. A comprehensive analysis of these indices indicates that the microbial richness and diversity in the SS samples were relatively higher.

#### 3.4.3. Characteristics of Soil Community Changes at Different Depths of Soil-like Substrate

**Figure 9:**
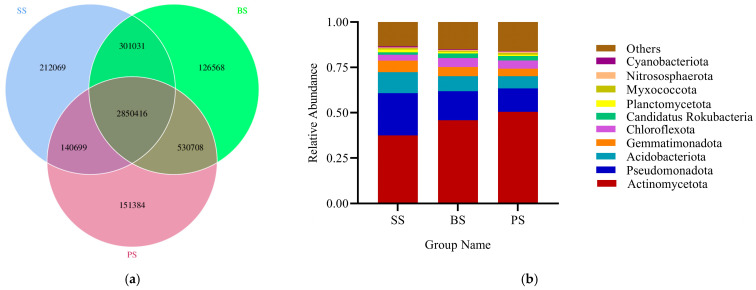
Soil microbial community composition at different depths in the soil-like substrate technology zone. (**a**) Venn Diagram of Gene Numbers; (**b**) The relative abundance of bacterial communities at the phylum level. The others are all microorganisms with an abundance below ten, including both identified and unidentified taxonomic units.

From the Venn diagram, it can be concluded that the total number of genes shared among the soil samples from different layers is 2,850,416; the unique gene counts for SS, BS, and PS are 212,069, 126,568, and 151,384, respectively (Figure 9a). At the phylum level, the microbial community composition of the soil-like substrate at different depths is essentially consistent, but there are significant differences in community structure and relative abundance (Figure 9b). Among the top three most abundant phyla, *Actinomycetota* is the dominant phylum, accounting for 37.46–50.46%. In SS, the relative abundance of *Actinomycetota* decreased by 18.31% and 25.76% compared to BS and PS, respectively. For *Pseudomonadota*, the relative abundance in SS increased by 46.08% and 80.77% compared to BS and PS, respectively. For *Acidobacteriota*, the relative abundance in SS increased by 40.45% and 72.09% compared to BS and PS, respectively.

#### 3.4.4. LEfSe Analysis of Differential Species in Soil-like Substrate at Different Depths

**Figure 10:**
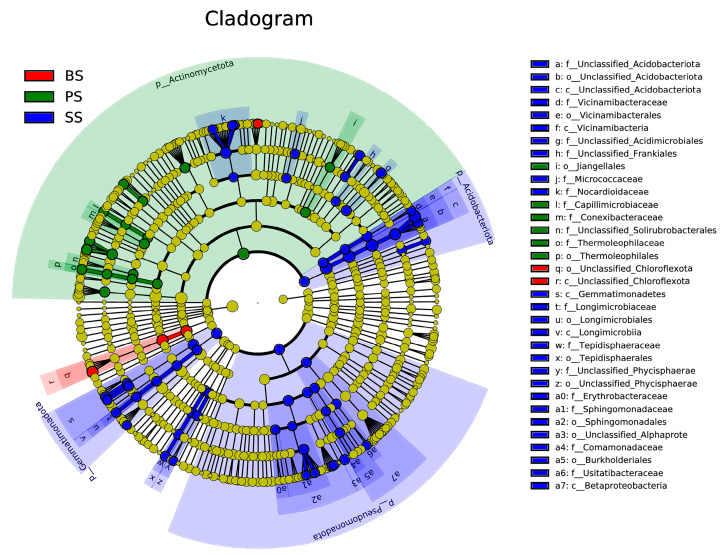
Cladogram of differentially abundant species from LEfSe analysis at different depths in the soil-like substrate technology zone. The circles radiating from the inside out in the figure represent the taxonomic levels from phylum to genus (or species). At each taxonomic level, each small circle represents a classification at that level, with the diameter of the small circle being proportional to its relative abundance. The coloring principle is as follows: species with no significant differences are uniformly colored yellow, while biomarker species with differences are colored according to their respective groups. Blue nodes indicate microbial groups that play an important role in the SS group, green nodes indicate microbial groups that play an important role in the PS group, and red nodes indicate microbial groups that play an important role in the BS group. The species names represented by the English letters in the figure are displayed in the legend on the right.

After LEfSe analysis, differential species at the family or order level among different soil layers of soil-like substrate at various depths were identified (Figure 10). Differential species were colored according to different groupings, with LDA > 4 used as the screening criterion. The following 16 strains were significantly enriched in the SS group: *Nocardioidaceae*, *Micrococcaceae*, *Unclassified-Frankiales*, *Unclassified-Acidimicrobiales*, *Vicinamibacteria*, *Acidobacteriota*, *Usitatibacteraceae*, *Comamonadaceae*, *Burkholderiales*, *Alphaproteobacteria*, *Betaproteobacteria*, *Erythrobacteraceae*, *Sphingomonadaceae*, *Tepidisphaeraceae*, *Unclassified-Phycisphaerae*, and *Gemmatimonadetes*. The following 2 strains were significantly enriched in the BS group: *Chloroflexota* and *Kribbella*. The following 5 strains were significantly enriched in the PS group: *Jiangellales*, *Conexibacteraceae*, *Capillimicrobiaceae*, *Thermoleophilaceae*, and *Unclassified-Solirubrobacter*. The enriched microbial communities mainly belong to the phyla *Actinomycetota*, *Pseudomonadota*, *Acidobacteriota*, *Gemmatimonadota*, and *Chloroflexota*. These microbial groups play important roles in their respective soil categories and show significant differences.

#### 3.4.5. Analysis of Functional Characteristics of Soil Microbial Communities at Different Depths of Soil-like Substrate

**Figure 11:**
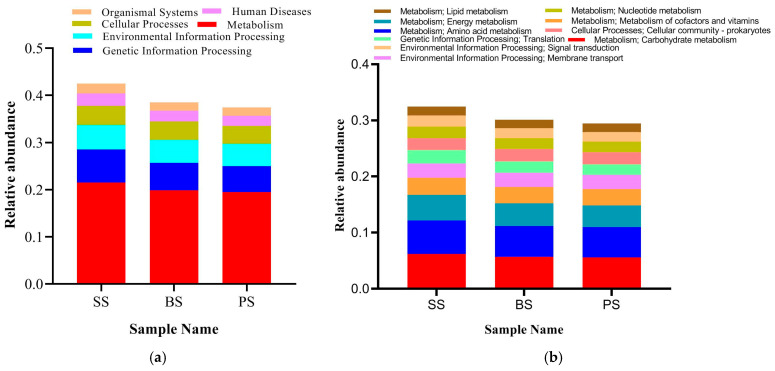
Sample functional annotation profiles at KEGG level 1 (**a**) and level 2 (**b**) at different depths in the soil-like substrate technology zone.

Based on the KEGG database, gene function prediction was performed on the non-redundant gene sets of soil microbes from different depths of soil-like substrate. In terms of biological metabolic pathways, at the level 1 category (Figure 11a), the three different soil depths showed differences in the relative abundance of six major metabolic pathways. The SS layer had higher relative abundances of Metabolism and Gene information processing pathways compared to the BS and PS treatments. At the level 2 category, the top ten metabolic pathways with the highest relative abundance in functional annotation for the three soil samples were Carbohydrate metabolism, Amino acid metabolism, Energy metabolism, Metabolism of cofactors and vitamins, Membrane transport, Translation, Cellular community—prokaryotes, Nucleotide metabolism, Signal transduction, and Lipid metabolism. Among these, the relative abundances of Carbohydrate metabolism and Amino acid metabolism in SS were significantly higher than those in BS and PS. Specifically, in Carbohydrate metabolism, SS had increases of 8.75% and 10.99% compared to BS and PS, respectively; in Amino acid metabolism, SS had increases of 8.89% and 10.92% compared to BS and PS, respectively (Figure 11b). A comprehensive analysis at both level 1 and level 2 shows that, although the metabolic pathways detected in soils at different depths are essentially consistent, there are still significant differences in the abundance of these metabolic pathways.

### 3.5. Relationships Between Soil Microbial Community Structure and Nutrient Content, Enzyme Activity

**Figure 12:**
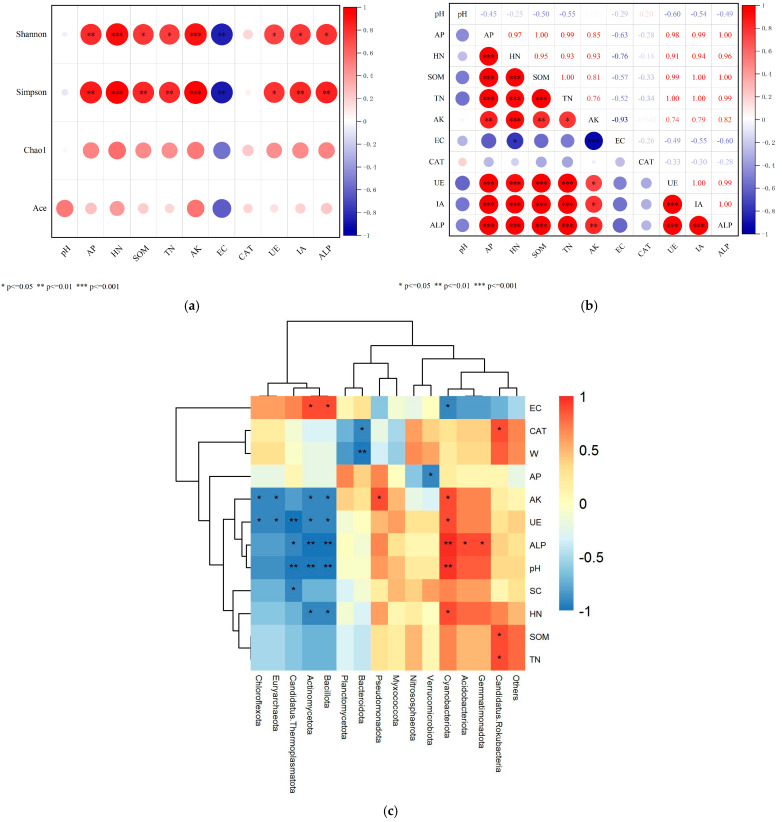
Association of soil microbial community composition with nutrient contents and enzyme activities at different depths in the soil-like substrate technology zone. (**a**) Correlation analysis of alpha diversity indices with nutrient contents and enzyme activities; (**b**) Correlation analysis between nutrient contents and enzyme activities; (**c**) Heatmap of correlation between soil environmental factors and microbial community composition; Asterisks denote significance levels: * *p* < 0.05; ** *p* < 0.01; *** *p* < 0.001. The different colored squares represent the associations between microbial communities and nutrient content and enzyme activity. The darker the color, the more significant the association. Red represents positive correlations, while blue represents negative correlations. The different branches represent the clustering of microbial communities. In (**a**,**b**), circle size indicates association strength.

The results of Pearson correlation analysis showed that, in terms of soil properties and soil microbial diversity, AP, HN, SOM, TN, AK, UE, IA, and ALP were significantly positively correlated with the Shannon and Simpson indices, while EC was significantly negatively correlated with the Shannon and Simpson indices (Figure 12a). Regarding the relationship between soil physicochemical properties and enzyme activity, AP, HN, SOM, TN, and AK were significantly positively correlated with UE, IA, and ALP, while EC was significantly negatively correlated with HN and AK (Figure 12b). Spearman correlation analysis indicated significant relationships between environmental factors and microbial communities (Figure 12c).

## 4. Discussion

### 4.1. Effects of Soil-like Substrate Improvement Technology on Soil Nutrient Content, Enzyme Activity, and Microbial Community Structure

The soil microbiome plays a key role in promoting nutrient cycling, enhancing soil carbon and nitrogen fixation capabilities, and maintaining fertility [28]. The TN, HN, AP, AK, and SOM contents in the RLS were significantly higher than those in CK (*p* < 0.05), which are direct factors for the improvement of soil fertility. Urease (UE) is a key enzyme in the nitrogen cycle, and its enhanced activity is usually closely related to the enrichment of ammonifying and nitrifying bacteria, such as *Betaproteobacteria* in the phylum *Proteobacteria* [29]. The increases of 135.29% and 130.68% in TN and HN contents in the soil of the RLS area are directly related to the 115.15% increase in urease (UE) activity. At the same time, the significant increases of 54.48% and 15.97% in the relative abundances of *Deltaproteobacteria* and *Betaproteobacteria* in the phylum *Proteobacteria* further confirm the close correlation between nutrients, enzyme activity, and microorganisms.

Soil pH is a key environmental factor affecting microbial diversity and community structure [30,31]. In this study, the soil pH in RLS was significantly higher than that in CK, indicating that the soil improvement measures significantly increased the pH value, which also confirms that pH is one of the important factors driving changes in microbial communities [32]. In the RLS soil samples, the activity of CAT increased by 13.14%, which may be due to the synergistic action of *Actinomycetes* and *Proteobacteria*. *Actinomycetes* can secrete CAT to decompose oxidative stress products [33], and some strains of *Proteobacteria* also have high CAT activity [34]. In addition, the increase of 134.76% in IA activity may be related to the enrichment of carbohydrate-metabolizing bacteria (such as *Gemmatimonadetes*), whose abundance increased by 27.66% in RLS and was significantly positively correlated with IA activity (*p* < 0.05).

### 4.2. Analysis of Soil Nutrients, Enzyme Activity, and Microbial Community Structure and Functional Characteristics at Different Depths of Soil-like Substrate Technology

To further elucidate the mechanisms of soil improvement by soil-like substrate technology, this study focused on the changes in soil nutrient content, enzyme activity, microbial community structure, and functional predictions at different depths in the experimental area. The results showed that the contents of nitrogen, phosphorus, potassium, and organic matter in the surface soil (SS) were significantly higher than those in the deeper soil layers, and the activities of corresponding soil enzymes (such as UE, ALP, and IA) also reached their peaks. These results indicate that the nutrients and enzyme activities in the surface soil are one of the factors affected by the soil improvement technology of soil-like substrate. These factors affect the structure and functions of microbial communities, thereby influencing the ecological functions of the soil. Liu J.A. et al. [35] reported that a high ratio of organic fertilizer substitution significantly reduced soil acidification, improved soil nutrient status, and enhanced soil urease and catalase activities (*p* < 0.05). Chen Y.Q. et al. [36] found that vegetation restoration significantly improved soil physicochemical properties and the overall soil quality index (SQI), and Strong positive correlations existed between microbial biomass and the SQI. At the same time, the high biological activity in the soil surface may be closely related to the presence of vegetation. Vegetation can affect the availability of water, nutrient input, and enzyme activity through the rhizosphere. Soil enzymes mainly come from microorganisms, plant roots, and animals. The input of organic matter directly stimulates the activity of microorganisms, thus becoming one of the most critical driving factors affecting soil enzyme activity [37]. Plant roots can selectively enrich specific microbial communities [38], which help plants absorb nutrients through symbiotic or mutualistic relationships, while decomposing organic matter and releasing nutrients to further improve soil fertility [39]. Vegetation can also regulate soil moisture conditions by increasing the water retention capacity of the rhizosphere [40]. Carbon input from living roots is 2 to 13 times more efficient than that from aboveground litter in stabilizing soil structure [41]. The main vegetation in areas improved by soil-like substrate technology is herbaceous plants and small shrubs, which have shallow roots that can provide richer living and activity conditions for soil surface microorganisms. Therefore, the high biological activity of the surface soil is not only determined by nutrients and enzyme activity, but the rhizosphere processes of vegetation also play a key role. These processes together promote the diversity of soil microbial communities, thereby affecting the ecological functions of the soil. Future research should further explore the interaction between vegetation rhizosphere processes and soil improvement technologies to better understand the mechanism of soil-like substrate improvement technology.

In the SS layer, microbial alpha diversity analysis (Shannon and Simpson indices) and the abundance of carbohydrate metabolism pathways were significantly higher than those in the deeper BS and PS layers, which were positively correlated with the high contents of nitrogen, phosphorus, potassium, and organic matter in the SS layer. The phylum *Actinomycetota*, as the dominant bacterial group, is primarily due to the oligotrophic adaptation, strong metabolic capabilities, and stress resistance of actinomycetes [42], which can participate in carbon cycling and symbiotic interactions [43]. However, in this study, the relative abundance of *Actinomycetota* in the SS surface layer was lower, decreasing by 18.31% and 25.76% compared to BS and PS, respectively. This may be due to the gradual recovery of vegetation after soil improvement, with the high contents of nitrogen, phosphorus, potassium, and organic matter in the surface layer and the corresponding high enzyme activities leading to significant increases in the abundance and diversity of bacteria, fungi, and other microorganisms, thereby weakening the competitive advantage of actinomycetes in the surface soil [44]. Meanwhile, vegetation recovery altered the physicochemical properties of the soil and the interactions within microbial communities [45], and the rapid changes in soil environmental conditions were not conducive to the growth of actinomycetes.

The phyla *Pseudomonadota* and *Acidobacteriota* play key roles in nitrogen and phosphorus cycling, with *Pseudomonadota* being particularly closely related to the activities of urease and phosphatase in the soil [46]. In the SS layer, where the indicators of nutrient content (nitrogen, phosphorus, potassium, and organic matter) are the highest and the activities of UE and ALP also reach their peaks, these critical environmental factors provide abundant substrates for *Pseudomonadota* and *Acidobacteriota* to participate in nitrogen and phosphorus cycling. At a pH of 8.6–8.76, phosphorus undergoes reverse transformation. However, *Pseudomonas* species with high abundance can secrete alkaline phosphatase. They can also dissolve the insoluble phosphorus in the soil by secreting organic acids and other substances, thereby increasing the content of available phosphorus in the soil. Additionally, *Pseudomonadota* interacts with plant root exudates to modulate microbial community structure [47]. After soil-like substrate improvement, the rich root exudates from vegetation in the surface layer serve as a nutrient source, promoting the increased relative abundance of these two bacterial groups. LEfSe analysis revealed that the enrichment of *Burkholderiales* and *Sphingomonadaceae* in the SS layer is related to their versatile metabolic capabilities, which are associated with higher UE and ALP activities [48].

## 5. Conclusions

The findings indicated that the soil-like substrate technology, by modulating soil pH and nutrient content, significantly altered the structure of microbial communities, with Actinobacteria and Pseudomonadota emerging as the core groups driving nutrient cycling. The enrichment of these microbial groups was closely associated with the increased activities of key enzymes such as urease (UE) and alkaline phosphatase (ALP) in the soil. The high metabolic activity in the surface soil was closely related to the input of easily decomposable carbon, while the deep microbial communities adapted to resource-limited environments. Soil-like substrate technology provides a practical basis for the coordinated restoration of the “soil-vegetation-microorganism” system on the Loess Plateau. Future research could focus on the targeted regulation of functional strains to further optimize ecological restoration efficiency.

## Figures and Tables

**Table 1:** Nutrient contents in the soil of the control and treatment areas.

Sample Name	pH	TN (g·kg^−1^)	SOM (g·kg^−1^)	AP (mg·kg^−1^)	HN (mg·kg^−1^)	AK (mg·L^−1^)	EC (mS·cm^−1^)
CK	7.94 b ± 0.02	3.7 b ± 0.01	6.66 b ± 0.22	7.8 b ± 0.12	24.80 b ± 0.80	11.087 b ± 0.19	1.88 a ± 0.05
RLS	8.41 a ± 0.05	8.8 a ± 0.01	13.5 a ± 0.10	12.1 a ± 0.12	57.21 a ± 4.02	18.559 ^a^ ± 0.23	0.329 b ± 0.003

Mean ± standard deviation, *n* = 3; Different lowercase letters indicate significant differences at *p* < 0.05 between treatments.

**Table 2:** The abundance percentage of microbial communities in CK and RLS samples at the phylum and class levels.

Phylum	Class	Microbial Abundance of the Sample		Increase or Decrease
		CK	RLS	In Percentage (%)
*Actinobacteria*	*Rubrobacteria*	0.012	0.019	37.19
*Proteobacteria*	*Deltaproteobacteria*	0.004	0.008	54.48
*Actinobacteria*	*Actinomycetes*	0.297	0.165	−44.45
*Proteobacteria*	*Betaproteobacteria*	0.04	0.048	15.97
*Gemmatimonadetes*	*Gemmatimonadetes*	0.008	0.011	27.66
*Actinobacteria*	*Thermoleophilia*	0.076	0.088	13.29
*Proteobacteria*	*Alphaproteobacteria*	0.072	0.049	−31.33

**Table 3:** Detection of Soil Nutrient Content at Different Depths of Soil-Like Substrate.

Sample Name	pH	TN (g·kg^−1^)	SOM (g·kg^−1^)	AP (mg·kg^−1^)	HN (mg·kg^−1^)	AK (mg·L^−1^)	EC (mS·cm^−1^)
SS	8.6 ^b^ ± 0.01	1.29 ^a^ ± 0.001	22.40 ^a^ ± 0.35	79.9 ^a^ ± 2.02	99.38 ^a^ ± 0.93	27.64 ^a^ ± 0.63	0.188 ^b^ ± 0.01
BS	8.76 ^a^ ± 0.03	0.9 ^c^ ± 0.0004	14.2 ^b^ ± 0.13	7.5 ^b^ ± 0.16	73.56 ^b^ ± 3.24	21.72 ^b^ ± 0.39	0.195 ^b^ ± 0.01
PS	8.6 ^b^ ± 0.04	0.93 ^b^ ± 0.001	14.4 ^b^ ± 0.16	3.5 ^c^ ± 0.24	63.79 ^c^ ± 2.32	13.84 ^c^ ± 0.11	0.243 ^a^ ± 0.01

Mean ± standard deviation, *n* = 3; Different lowercase letters indicate significant differences at *p* < 0.05 between treatments.

## Data Availability

The original contributions presented in this study are included in the article. Further inquiries can be directed to the corresponding authors.

## Abbreviations

The following abbreviations are used in this manuscript:

CK	The control zone
RLS	−20 cm depth layer of the soil-like substrate
LS	The soil-like substrate technology zone
SS	Soil-like substrate of the surface layer (0–6 cm)
BS	Soil-like substrate of the substratum layer (6–12 cm)
PS	Soil-like substrate of the vegetation mat substrate layer (12–20 cm)
TN	Total nitrogen
HN	Hydrolyzable nitrogen
AP	Available phosphorus
AK	Available potassium
SOM	Organic matter
EC	Electrical conductivity
UE	Urease
CAT	Catalase
ALP	Alkaline phosphatase
IA	Invertase
KEGG	Kyoto Encyclopedia of Genes and Genomes
